# Running interval training combined with blood flow restriction increases maximal running performance and muscular fitness in male runners

**DOI:** 10.1038/s41598-022-14253-3

**Published:** 2022-06-15

**Authors:** Yun-Tsung Chen, Yao-Yi Hsieh, Jen-Yu Ho, Chien-Chang Ho, Tung-Yi Lin, Jung-Charng Lin

**Affiliations:** 1 Department of Physical Education and Sport Sciences, National Taiwan Normal University, Taipei, Taiwan; 2 Department of Athletic Performance, National Taiwan Normal University, Taipei, Taiwan; 3 Department of Physical Education, Fu Jen Catholic University, New Taipei City, Taiwan; 4 Institute of Traditional Medicine, National Yang Ming Chiao Tung University, Taipei, Taiwan; 5 Graduate Institute of Sport Coaching Science, Chinese Culture University, Taipei, Taiwan

**Keywords:** Skeletal muscle, Blood flow, Respiration

## Abstract

We investigated the effects of 8 weeks (3 days per week) of running interval training (RIT) combined with blood flow restriction (RIT-BFR) on the maximal running performance (RPmax), isokinetic muscle strength, and muscle endurance in athletes. Twenty endurance-trained male runners were pair-matched and randomly assigned to the RIT-BFR and RIT groups. The RIT-BFR group performed RIT (50% heart rate reserve, 5 sets of 3 min each, and 1-min rest interval) with inflatable cuffs (1.3× resting systolic blood pressure), and the RIT group performed the same RIT without inflatable cuffs. RPmax, isokinetic muscle strength, and muscle endurance were assessed at pre-, mid-, and post-training. Compared with the RIT group, the RIT-BFR group exhibited a significantly (*p* < 0.05) greater increase in RPmax, isokinetic knee extensor and flexor strength, and knee extensor endurance after 24 training sessions. These results suggested that RIT-BFR may be a feasible training strategy for improving muscular fitness and endurance running performance in distance runners.

## Introduction

High-intensity interval training (HIIT) is a time-efficient training strategy for improving aerobic capacity (i.e., maximal oxygen uptake [${\dot{\text{V}}\text{O}}_{2}$max]) and aerobic endurance performance (e.g., running and cycling time trials) in contrast to high-volume endurance training in athletes^1–3^. HIIT consists of repeated high-intensity exercise bouts (at ≥ 90% ${\dot{\text{V}}\text{O}}_{2}$max for < 45 s or 2–4 min intervals) interspersed with passive rest or low-intensity exercise recovery periods (≤ 2 min)^4^. Such improvements in endurance performance have been suggested to be due to HIIT-induced increases in mitochondria content, capillary density, oxidative enzyme activity, and acid-buffering capacity^3,5^. However, HIIT also induces elevated levels of catabolic hormones (e.g., cortisol)^6–8^, which may suppress muscle protein synthesis, thereby reducing muscle fiber hypertrophy and muscle strength development after training^9^.

Low-intensity aerobic training (20%–40% ${\dot{\text{V}}\text{O}}_{2}$max for 10–15 min) combined with blood flow restriction (BFR) is a novel training method for improving aerobic endurance performance, achieving muscle hypertrophy, and enhancing muscle strength in young adults^10,11^. BFR (pressure: 160–230 mmHg) is achieved by applying inflatable cuffs to the proximal lower extremity during walking or cycling training. The strategy has been suggested to partially restrict venous and arterial blood flow without changing the muscle damage markers such as creatine kinase and myoglobin. Barjaste et al.^12^ reported that in healthy men, an acute walking exercise with BFR (40% ${\dot{\text{V}}\text{O}}_{2}$max, 5 sets of 2 min each, 1-min rest interval, 200 mmHg) increased the activity of the cell signaling network responsible for muscle protein expression, mitochondrial biogenesis, and angiogenesis as well as increased the responses of anabolic hormones, such as growth hormone and insulin-like growth factor-1. These are probable mechanisms underlying the improvement in aerobic endurance performance and muscle hypertrophy. Studies examining the effects of BFR have mainly focused on aerobic training in untrained individuals^10,11,13^. However, Park et al. reported that low-intensity walking interval training combined with BFR (40% ${\dot{\text{V}}\text{O}}_{2}$max, 5 sets of 3 min each, 1-min rest interval, 160–220 mmHg) increased aerobic capacity but not muscle strength in male basketball players^14^. Similarly, Held et al. observed that rowing interval training (intensity < 2 mmol/L, 2 10-min sets, 10-min rest interval) combined with BFR increased aerobic capacity but not squat performance in elite rowers^15^. These findings demonstrate that the intensity of aerobic training for power- and endurance-trained athletes may require an increase (e.g., to > 2 mmol/L or through running) to improve both aerobic performance and muscle strength.

Notably, endurance runners with good muscular fitness (encompassing both muscle strength and muscle endurance) tend to have not only a lower risk of muscle injury^16^ but also a higher maximal running performance (known as peak treadmill running velocity), which is a good predictor of endurance running performance (e.g., in 10, 21.1, and 42.2 km of running)^17,18^. It has reported that in running the role of the hip extensors especially the hamstring muscles is the essential for producing hip power and pushing the body forward with increasing running speed^19^. In addition, the knee extensor is to create high joint stiffness before and during the contact phase, resulting spring-like behavior of muscles (stretch–shortening cycle) and transmit the work done by hip extensors better, thereby propelling the body forward more effectively^19,20^. Moreover, a significant correlations have been reported between the 35-m sprinting time and isokinetic hip extensor, knee extensor and flexor strength at velocities of 60, 150, 180 and 240°/s for track sprinters and rugby players^21^. Compared with the low-speed (60°/s) muscle contractions, the relationships were improved when the isokinetic knee extensor and flexor strength were measured at high-speed (180 or 240°/s) muscle actions^21^. Therefore, athletes with greater velocity‐specific muscle strength may have beneficial effects on running performance.

However, the short period of aerobic training combined with BFR on maximal running performance (RPmax), velocity‐specific muscle strength and endurance performance in runners require further investigation. Furthermore, we believe that previous studies focused on changes in hormones, ${\dot{\text{V}}\text{O}}_{2}$max and isokinetic (60°/s) muscle strength are not enough to explain the great effects of BFR training strategy. Thus, in the current study, we further analyzed the other part of the data obtained from the same study design^22^. The purpose of this study was to investigate the effects of 8 weeks of RIT-BFR on RPmax, isokinetic (180°/s) muscle strength, and endurance performance in male runners.

## Methods

### Participants

Twenty male runners (mean ± standard deviation [SD]; age, 21.6 ± 2.2 years [19–25 years]; height, 177.6 ± 6.8 cm; body mass, 69.0 ± 6.2 kg; training year, 7.1 ± 2.1 years; SBP, 121.6 ± 5.7 mmHg) participated in this study (Table 1). The athletes were in the off-season phase of their training during the study. All subjects were well informed about the experimental methods, procedures, and possible benefits and risks, and they provided informed consent before participation. Eligibility criteria included: (a) male adults (≥ 18 years old) who had ≥ 3 years of cardiopulmonary endurance training experience (1500 and 5000 m runners, triathlon and marathon athletes); (b) subjects who have competed in National Intercollegiate Athletic Games. Exclusion criteria included: (a) subjects who were hypertensive (> 130/80 mmHg); (b) subjects who had any known cardiovascular disease or muscle injuries that may hinder them from performing RIT and exercise testing; (c) subjects who were taking growth hormone, testosterone or other similar anabolic hormonal pharmaceuticals.

**Table 1:** Subject anthropometrics.

	RIT-BFR	RIT	*p* value
Age (years)	21.5 ± 2.2	21.6 ± 2.1	0.90
Height (cm)	175.0 ± 6.8	180.1 ± 6.7	0.11
Body mass (kg)	66.3 ± 4.4	71.7 ± 7.9	0.08
Training year (years)	7.1 ± 2.4	6.9 ± 1.6	0.78
${\dot{\text{V}}\text{O}}_{2}$max (ml/kg/min)	64.3 ± 4.7	60.3 ± 5.4	0.10
HRrest (beats/min)	66.1 ± 6.8	66.5 ± 5.9	0.86
HRmax (beats/min)	197.2 ± 6.7	204.8 ± 7.8	0.05
SBPrest (mmHg)	118.5 ± 6.4	124.8 ± 4.9	0.14

### Experimental design and procedures

**Figure 1:**
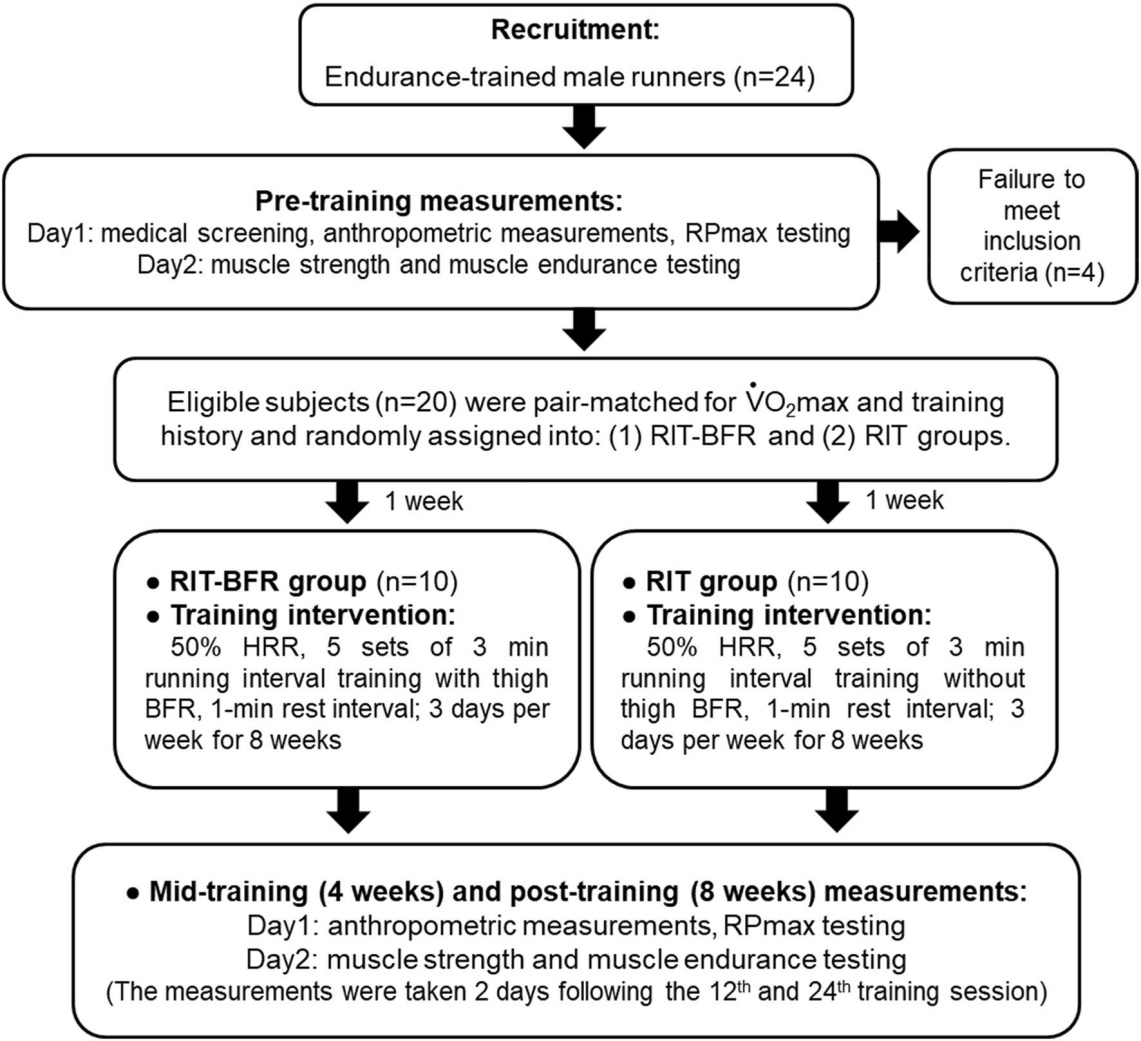
Overview of research design. RIT-BFR: running interval training combined with blood flow restriction group; RIT: running interval training only group; anthropometric measurements: height, body mass and resting systolic blood pressure; RPmax: maximal running performance.

To examine the effects of 8 weeks of RIT combined with blood flow restriction on RPmax, high-speed muscle strength and muscle endurance of endurance-trained athletes, we matched up (by ${\dot{\text{V}}\text{O}}_{2}$max and training history) and assigned 20 male runners into 2 experimental groups: (a) RIT with BFR group (RIT-BFR, n = 10) and (b) RIT without BFR group (RIT, n = 10). All subjects received 24 training sessions over 8 weeks; each training session was conducted between 08:00 and14:00 on each training day at least 48 h apart. Muscular fitness and RPmax were measured at pre-, mid- and post-training as shown in Fig. 1.

One week before the first training session, subjects visited the laboratory twice for pre-training measurements. Each subject received medical questionnaire and anthropometric measurements. Subjects then performed an RPmax test. Following a 24 h rest after RPmax testing, subjects performed isokinetic muscle strength and muscle endurance tests. On the day of each training session, all subjects performed 5 sets of 3 min running at 50% HRR on a treadmill, with a 1-min rest interval. Subjects in the RIT-BFR group performed the RIT with inflatable cuffs on each thigh. Subjects in the RIT group performed the same RIT without inflatable cuffs.

Subjects performed the same RPmax, isokinetic muscle strength and muscle endurance tests as pre-training measurements during the mid- and post-training (4 weeks and 8 weeks), 2 days following the 12th and 24th training sessions. They were asked to refrain from caffeine, alcohol and strenuous exercise for at least 48 h before RPmax and muscular fitness tests. Subjects were verbally encouraged to exert maximum effort during these tests.

### Anthropometric measurement

Height was measured using a digital stadiometer to the nearest 0.1 cm (Jenix DS-102; Dong Sang Jenix Co., Ltd., Seoul, South Korea). Body mass was measured through bioelectrical impedance analysis to the nearest 0.1 kg (InBody 720; Biospace Co., Ltd., Seoul, Korea). After 15‒20 min of rest, subjects performed resting heart rate (HRrest) and brachial SBP measurement using an automatic blood pressure monitor (HEM-7000-C1; OMRON Healthcare Co., Ltd., Kyoto, Japan). SBP was taken twice in a seated position, and if the values were not within 5 mmHg, a third measurement was conducted^23^. The closest two values were averaged and used as a reference. They were asked to have fasted for at least 3 h and maintained normal hydration before anthropometric measurements.

### Running interval training intervention

The training session was conducted 3 days per week for 8 weeks in both groups. All subjects performed 5 sets of 3 min running at 50% HRR running speed on a treadmill (Mercury COS10198; h/p/cosmos, Nussdorf-Traunstein, Germany) with 1-min rest between sets. This protocol was selected on the basis of the previous studies^14,24^. The running speed was set at a predetermined training intensity of 50% HRR, and the mean treadmill speed across all 24 training sessions on RIT-BFR and RIT group were 10.6 km/h and 10.3 km/h, respectively. The maximal HR (HRmax) and HRrest during the RPmax test were used to determine the HRR for each subject. The 50% HRR was estimated based on the equation: (HRmax – HRrest) × 50% + HRrest.

### Blood flow restriction

Subjects in the RIT-BFR group wore inflatable cuffs (14.2 cm × 67.5 cm, CK-113; Spirit, Taipei, Taiwan) on the most proximal portion of each thigh (inguinal fold region) during training sessions. Cuffs were applied by the same investigator to maximize intra-rater reliability of the pressure, and the intraclass correlation coefficient (ICC) was 0.86. A cuff pressure of 1.3 times resting SBP was selected, which increased muscle activation and metabolic stress without changing inflammation markers such as high-sensitivity C-reactive protein^24,25^. The cuff pressure was 154 ± 6 mmHg (1.3 times SBP), and the cuff remained inflated during the rest periods between sets. The cuffs were wrapped and inflated 10 s before each training session and released immediately after the fifth bout of running. The total time of cuff inflation was 19.5 ± 0.5 min. Subjects in the RIT group received training sessions without the inflatable cuffs. During training sessions, HR and rating of perceived exertion (RPE) were recorded at each 3-min running bout in both groups.

### Maximal running performance testing

Subjects performed an incremental treadmill (Mercury COS10198; h/p/cosmos, Nussdorf-Traunstein, Germany) exercise test (Bruce protocol as shown in Table 2)^26^ to volitional exhaustion for determining ${\dot{\text{V}}\text{O}}_{2}$max, RPmax and HRmax. The expired respiratory gases were collected and measured using a breath-by-breath system (Vmax29; Sensor Medics Corporation, Yorba Linda, CA, USA). Breath-by-breath data were stationary time-averaged over 30 s and the highest averaged ${\dot{\text{V}}\text{O}}_{2}$ value attained during the exercise test was regarded as ${\dot{\text{V}}\text{O}}_{2}$max^27^. The criteria to evaluate ${\dot{\text{V}}\text{O}}_{2}$max were addressed in previous studies^14,28^: (1) no increase in oxygen uptake despite further increases in treadmill speed, (2) respiratory exchange ratio ≥ 1.10, (3) HRmax within 10 beats/min of the age-predicted maximum (220 − age), and (4) ratings of perceived exertion (Borg RPE 6–20 point scale) ≥ 17. Of these criteria, at least two have to be met to be considered as ${\dot{\text{V}}\text{O}}_{2}$max.

**Table 2:** Bruce protocol.

Stage	Minutes	Grade%	Speed (km/h)	METs
1	3	10	2.7	5
2	6	12	4.0	7
3	9	14	5.4	10
4	12	16	6.7	13
5	15	18	8.0	15
6	18	20	8.8	18
7	21	22	9.6	20

METs: metabolic equivalents.

Maximal running performance was calculated as follows^29^: RPmax = (speed × 0.2 + 0.9 × speed × grade) ÷ 0.2; speed (km/h) = speed of the last whole completed minute; grade is in decimal form, 12% grade is 0.12.

### Muscular fitness testing

Subjects performed isokinetic muscle strength and muscle endurance tests using a Biodex dynamometer (Biodex Medical Systems IV, Shirley, NY, USA). Subjects completed familiarization for isokinetic muscle strength and muscle endurance tests procedure prior to the measurements. Subjects sat upright on a chair with their dominant leg firmly attached to the lever of the dynamometer. The pivot point of the lever was accurately aligned with the rotational axis of the knee joint maintained at the same position during the test. During the muscle strength test, subjects performed 5 repetitions of maximal isokinetic knee extensions and flexions between a 0° and 90° range of motion at velocities of 180°/s; the highest peak torque was used for analysis^16^. A knee joint angle of 0° corresponded to full extension. The concentric hamstring to quadriceps (H/Q) ratio was calculated as follows: the peak torque of the knee flexor divided by that of the knee extensor^16^.

Following 5-min rest after strength testing, subjects performed an isokinetic knee extension and flexion endurance test comprising 50 repetitions within a 0°‒90°range of motion at the velocity of 180°/s. Muscle endurance was determined by the total work of the knee extensions and flexions^30^. The fatigue index was determined by the percentage decrease in the amount of work during the last 10 contractions compared with that during the initial 10 contractions^30^; it was calculated as 100% ‒ (last 10 work of knee extensions [flexions] ÷ initial 10 work of knee extensions [flexions]) × 100%.

### Heart rate and rating of perceived exertion measurement

HR and RPE were continuously monitored and measured during the RPmax testing and RIT sessions using telemetry (Polar RS800CX; Polar Electro Oy, Kempele, Finland) and the Borg RPE scale (a 15-point scale ranging from 6 to 20).

### Statistical analyses

All statistical analyses were performed using SPSS software (version 17.0; SPSS, Chicago, IL, USA). All data are expressed as mean ± SD. The dependent variables were performed using the Shapiro–Wilk normality test. Subject characteristics, running speed, HR and RPE of two groups were compared using independent *t*-test. Because variations in group mean values existed between groups at pre-training, the subsequent data were normalized by dividing the data by their pre-training values and multiplying by 100, thereby giving the pre-training value of 100% for normalized data. Two-way analysis of variance (time × group) with repeated-measures was used to compare the normalized RPmax, muscle strength and muscle endurance. The Bonferroni post hoc test was applied when a significant difference was observed. Partial eta squared (*η*^2^) was used as an effect size measurement for ANOVA, with *η*^2^ of 0.01, 0.06, and 0.14 representing small, medium, and large effect sizes, respectively^31^. Statistical significance was set at α = 0.05.

### Ethical approval

This study was conducted according to the guidelines of the declaration of Helsinki, and approved by the Research Ethics Committee of the National Taiwan Normal University (ID: 201501HM002). All methods were performed in accordance with the relevant guidelines and regulations.

## Results

### RIT-BFR increased HR and RPE during all 24 training sessions

Running speed did not differ (10.6 ± 1.1 km/h vs. 10.3 ± 0.9 km/h, *t* = 1.09, *p* > 0.05) between the RIT-BFR and the RIT groups. HR was significantly higher (12.7%) in the RIT-BFR group than in the RIT group (163 ± 11 beats/min vs. 145 ± 6 beats/min, *t* = 6.74, *p* < 0.05; 74% HRR vs. 57% HRR, *t* = 9.87, *p* < 0.05). Similarly, RPE was significantly higher (65.6%) in the RIT-BFR group than in the RIT group (15.3 ± 2.3 vs. 9.6 ± 2.1, *t* = 9.02, *p* < 0.05).

### RIT-BFR enhanced RPmax

**Figure 2:**
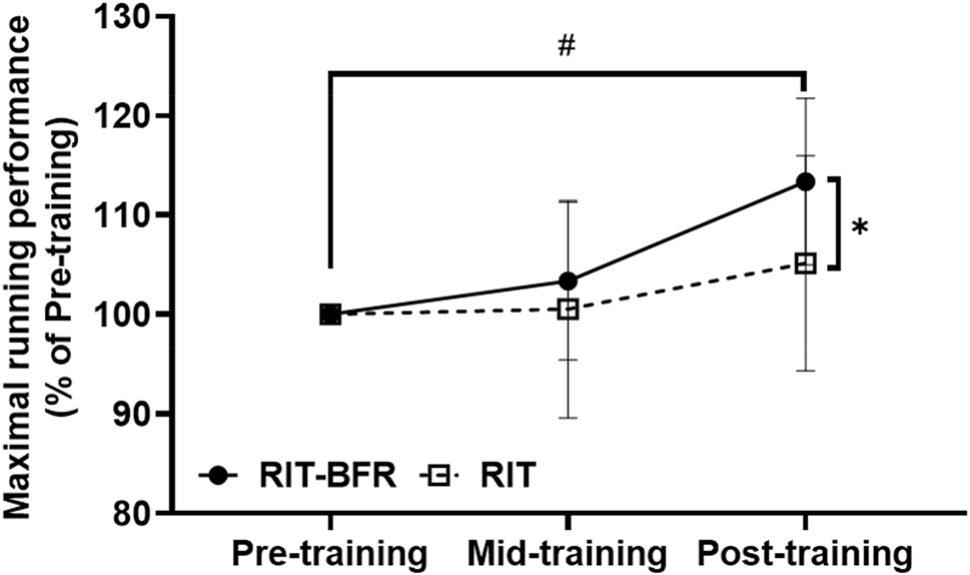
The percentage changes on maximal running performance after training. Statistical testing: Two-way ANOVA (repeated measures) with Bonferroni post hoc test; Data is displayed as mean ± SD. Significant differences were shown (^#^*p* < 0.05, compared with pre-training; and *p < 0.05, compared with the RIT group).

The raw data for RPmax as shown in Table 3. An interaction (*p* < 0.05) was observed for the normalized RPmax (Fig. 2). Post hoc analyses revealed that the post-training RPmax was significantly higher (*p* < 0.05) than the pre-training RPmax values in the RIT-BFR group. Additional analyses indicated that the normalized RPmax was significantly higher in the RIT-BFR group than in the RIT group (*F* = 7.99, *p* < 0.05, *η*^2^ = 0.30) after 8 weeks of training.

**Table 3:** Maximal running performance, muscle strength and endurance changes between RIT-BFR and RIT groups.

	Pre-training	Mid-training	Post-training
**Maximal running performance (km/h)**			
RIT-BFR	13.52 ± 1.85	13.82 ± 1.73	15.15 ± 1.08
RIT	13.00 ± 1.56	13.00 ± 1.56	13.59 ± 1.43
**Muscle strength 180°/s (Nm/kg)**			
Extensor			
RIT-BFR	1.61 ± 0.20	1.77 ± 0.26	1.88 ± 0.24
RIT	1.67 ± 0.25	1.71 ± 0.24	1.67 ± 0.22
Flexor			
RIT-BFR	1.17 ± 0.24	1.28 ± 0.24	1.39 ± 0.21
RIT	1.32 ± 0.21	1.34 ± 0.17	1.34 ± 0.20
H/Q ratio			
RIT-BFR	0.73 ± 0.12	0.74 ± 0.18	0.75 ± 0.14
RIT	0.81 ± 0.20	0.80 ± 0.19	0.82 ± 0.20
**Muscle endurance (J/kg)**			
Extensor			
RIT-BFR	73.37 ± 10.82	74.71 ± 14.50	79.87 ± 11.65
RIT	80.44 ± 9.51	80.09 ± 9.09	78.82 ± 7.12
Flexor			
RIT-BFR	57.64 ± 8.91	59.48 ± 10.75	62.87 ± 12.29
RIT	60.27 ± 9.37	62.05 ± 7.03	64.60 ± 8.58
**Fatigue index (%)**			
Extensor			
RIT-BFR	37.43 ± 10.18	44.81 ± 10.57	43.62 ± 8.55
RIT	50.72 ± 8.68	52.07 ± 10.76	52.43 ± 8.09
Flexor			
RIT-BFR	39.07 ± 9.51	43.82 ± 13.82	44.17 ± 9.16
RIT	35.11 ± 9.65	41.89 ± 11.10	44.14 ± 8.34

H/Q ratio: hamstring-quadriceps ratio.

### RIT-BFR enhanced muscle strength and muscle endurance

#### Isokinetic muscle strength

**Figure 3:**
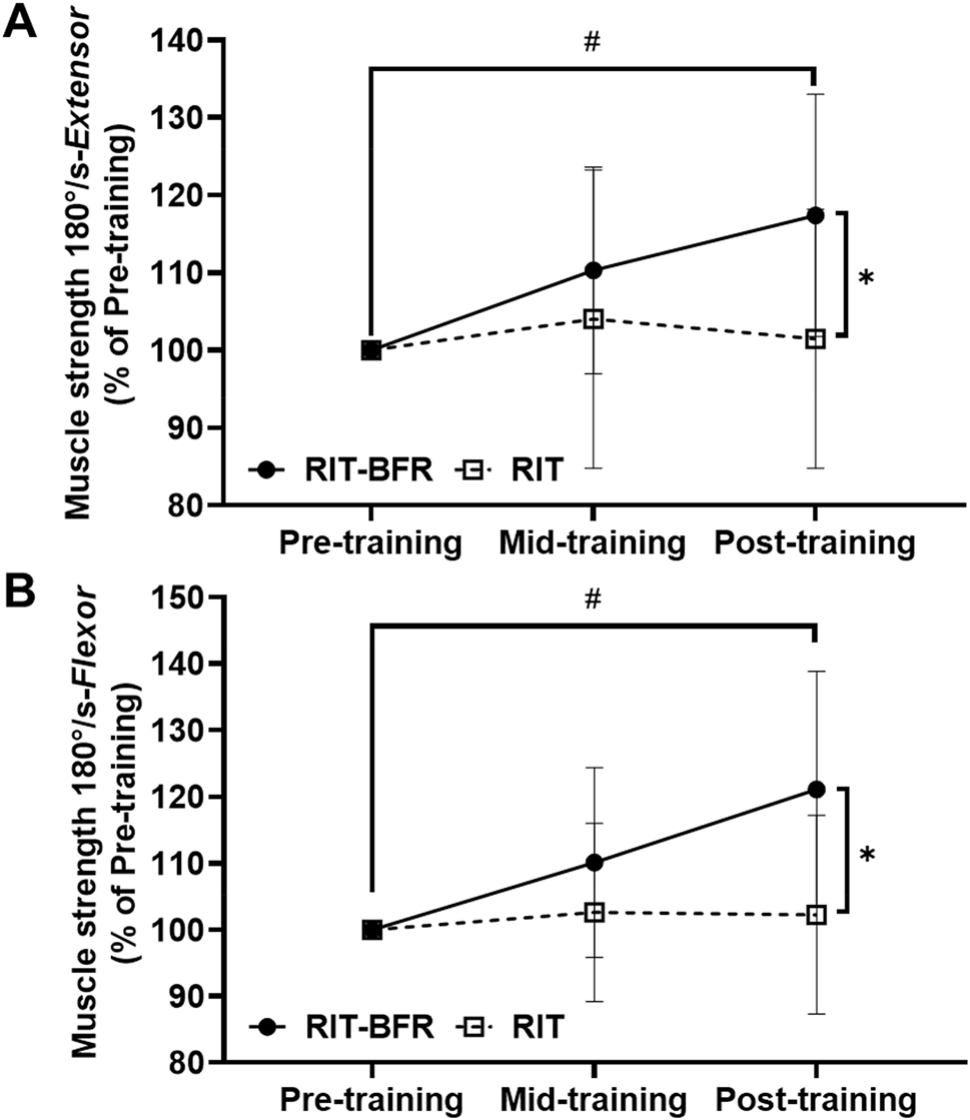
The percentage changes on muscle strength after training. (**A**) Isokinetic knee extensor strength; (**B**) isokinetic knee flexor strength; Statistical testing: Two-way ANOVA (repeated measures) with Bonferroni post hoc test; Data is displayed as mean ± SD. Significant differences were shown (^#^*p* < 0.05, compared with pre-training; and *p < 0.05, compared with the RIT group).

The raw data for isokinetic muscle strength as shown in Table 3. An interaction (*p* < 0.05) was observed for the normalized isokinetic knee extensor and flexor strength (Fig. 3). Post hoc analyses revealed that the post-training isokinetic knee extensor and flexor strength were significantly higher (*p* < 0.05) than the pre-training isokinetic knee extensor and flexor strength values in the RIT-BFR group. Additional analyses indicated that the normalized isokinetic knee extensor (*F* = 4.85, *p* < 0.05, *η*^2^ = 0.21) and flexor (*F* = 6.59, *p* < 0.05, *η*^2^ = 0.27) strength were significantly higher in the RIT-BFR group than in the RIT group after 8 weeks of training. However, no interaction (*p* > 0.05), time effects (*p* > 0.05), or group effects (*p* > 0.05) were observed for normalized H/Q ratio.

#### Isokinetic muscle endurance

**Figure 4:**
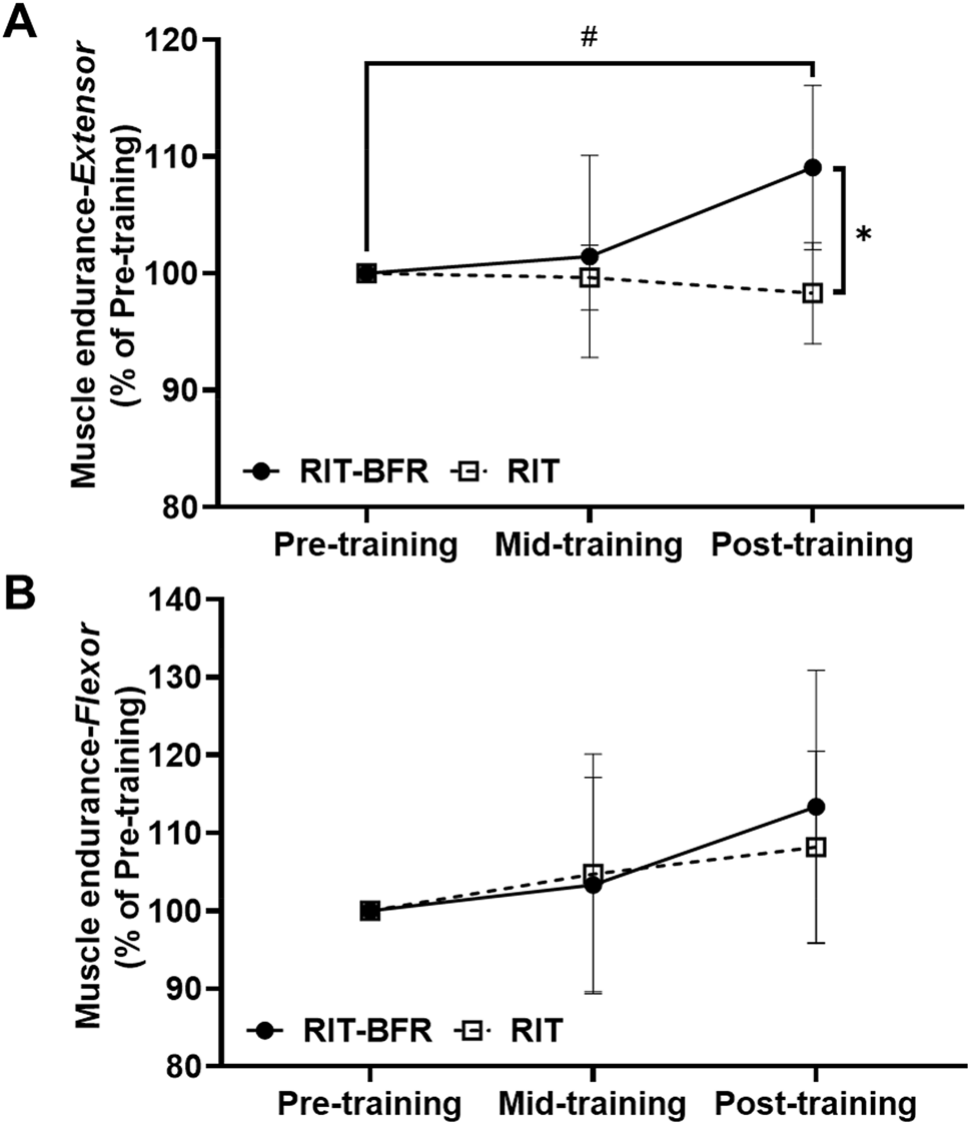
The percentage changes on muscle endurance after training. (**A**) Isokinetic knee extensor endurance; (**B**) isokinetic knee flexor endurance; Statistical testing: Two-way ANOVA (repeated measures) with Bonferroni post hoc test; Data is displayed as mean ± SD. Significant differences were shown (^*#*^*p* < 0.05, compared with pre-training; and *p < 0.05, compared with the RIT group).

The raw data for isokinetic muscle endurance as shown in Table 3. An interaction (*p* < 0.05) was observed for the normalized isokinetic knee extensor endurance (Fig. 4). Post hoc analyses revealed that the post-training isokinetic knee extensor endurance was significantly higher (*p* < 0.05) than the pre-training isokinetic knee extensor endurance values in the RIT-BFR group. Additional analyses indicated that the normalized isokinetic knee extensor endurance was significantly higher in the RIT-BFR group than in the RIT group (*F* = 16.96, *p* < 0.05, *η*^2^ = 0.49) after 8 weeks of training. However, no interaction (*p* > 0.05), time effects (*p* > 0.05), or group effects (*p* > 0.05) were observed for normalized isokinetic knee flexor endurance or fatigue index (Table 3).

## Discussion

This study further analyzed the other part of the data obtained from the same study design^22^, and try to show the impact of interval running combined with blood flow restriction on RPmax, high-speed muscle strength, and muscle endurance. The main finding of this study is that the RIT-BFR group had significantly greater RPmax, isokinetic (180°/s) knee extensor and flexor strength, and isokinetic knee extensor endurance compared with the RIT group after 8 weeks of training in endurance runners.

Our previous study showed that 8 weeks of RIT combined with BFR increased ${\dot{\text{V}}\text{O}}_{2}$max and maintained anabolic hormonal responses^22^. However, compared with the ${\dot{\text{V}}\text{O}}_{2}$max (*r* =  − 0.6 to  − 0.8), the RPmax exhibited a greater correlated (*r* =  − 0.9) with distance running performance in runners at 10–42.2 km^17,18^. A study indicated that 8 weeks of heavy resistance training (4–6 repetition maximum [RM]) increased neuromuscular adaptations (e.g., leg strength and vastus lateralis muscle activation) and may also exert beneficial effects on RPmax in endurance runners^17^. In the present study, we found that compared with RIT group, RIT-BFR group resulted in a greater increase in RPmax after 24 training sessions. In addition, training resulted in 17%, 21% and 9% increases in the isokinetic knee extensor and flexor strength, and isokinetic knee extensor endurance, respectively for RIT-BFR group. We speculated that the BFR training increased muscle hypertrophy and muscle oxidative (arteriovenous oxygen difference) and glycolytic capacity, which resulted in greater adaptations in muscular fitness^10,14,30,32^; this, in turn, may improve RPmax in endurance athletes. In contrast to our findings, those of Paton et al. indicated that running training (83% ${\dot{\text{V}}\text{O}}_{2}$max, [running at 8 intervals of 30 s each, 30-s rest interval] × 3 sets, 150-s rest interval) combined with BFR did not significantly increase RPmax after 8 training sessions (2 days per week for 4 weeks) in general population^33^. Therefore, having fewer training sessions may result in no improvement to endurance running capacity even if high-intensity running is combined with BFR.

Compared with the RIT group, the RIT-BFR group exhibited greater increases in isokinetic (180°/s) knee extensor and flexor strength after 24 training sessions. Our previous study observed that RIT-BFR group increased isokinetic (60°/s) knee extensor strength when compared with RIT group^22^. We suggested that RIT-BFR may be a practical training strategy for promoting isokinetic knee extensor strength at high- and low-speed muscle actions as well as increasing isokinetic knee flexor strength at high-speed muscle contraction in male runners. Similar studies have indicated that compared with cycling or walking alone, low-intensity cycling (40% ${\dot{\text{V}}\text{O}}_{2}$max) or walking (45% HRR) training combined with BFR increased knee extensor strength or flexor strength more substantially in young and older adults after 24–40 training sessions^10,32,34^. By contrast, in contrast to walking alone, walking training (40% ${\dot{\text{V}}\text{O}}_{2}$max) combined with BFR did not increase isokinetic (60°/s) knee extensor and flexor strength after 24 training sessions in basketball players^14^. We recommend increasing the aerobic training intensity and number of training sessions (e.g., ≥ 24 training sessions in which HRR or ${\dot{\text{V}}\text{O}}_{2}$max ≥ 50%) when BFR is implemented to improve muscle strength in power- and anaerobic-trained athletes.

Furthermore, a study revealed that track and field athletes may be at a 17-fold increased risk of hamstring injury if the H/Q peak torque (180°/s) ratio is less than 0.60^16^. In this study, the RIT-BFR group exhibited significant increases in both isokinetic knee extensor strength (17.4%) and flexor strength (21.1%) after 8 weeks of training. The RIT-BFR group maintained H/Q ratio (from 0.73 to 0.75) after training, suggesting that RIT with BFR not only increased hamstring and quadricep strength but also helped athletes maintain their strength balance.

Kacin and Strazar reported that in healthy men, BFR incorporated in resistance training with 15% maximal voluntary muscle contraction significantly improved muscle endurance and was associated with a significantly increased capacity for delivering oxygen to the muscles after 16 training sessions^35^. In addition, in elite rugby players, 50% 1RM resistance training combined with BFR significantly increased muscle endurance after 16 training sessions primarily because of muscle adaptations, such as increased acid-buffering capacity and oxidative energy metabolism^30^. Our study is the first to examine the effects of aerobic training combined with BFR on muscle endurance performance in endurance athletes. The results indicated that compared with the RIT group, the RIT-BFR group exhibited greater increases in isokinetic knee extensor endurance after 8 weeks of training. Although knee flexor endurance did not significantly differ between the groups, isokinetic knee flexor endurance tended to increase in both the RIT-BFR and RIT groups (9.3% and 8.2%, respectively) after 24 training sessions. However, neither training effects nor group differences were observed in isokinetic knee extensor and flexor fatigue indexes in the RIT-BFR or RIT groups. Therefore, the increase in knee extensor strength in the RIT-BFR group is the most reasonable explanation for the improved knee extensor endurance.

This study has some limitations. First, we did not measure the muscle tissue oxygenation index, oxidative energy metabolism, or muscle buffering capacity. Without these measurements, it may be difficult to elucidate the operating mechanisms of RIT-BFR with regard to effects on muscle endurance. Second, we did not establish a “BFR only” group. Without which the effectiveness of RIT-BFR in improving the muscular fitness and RPmax of runners cannot be thoroughly explored. Finally, our RPmax is a calculated performance variable by using running speed and grade during ${\dot{\text{V}}\text{O}}_{2}$max testing, and therefore this variable is not actual velocity. Further research on the effectiveness of RIT-BFR in enhancing maximal field running speed is warranted.

## Conclusion

This study demonstrated that 8 weeks of RIT-BFR increased maximal running performance, quadricep and hamstring strength, and quadricep endurance performance as well as preserved thigh strength balance in male runners. Combining previous and current studies, we fully explain that this special training strategy can bring excellent training benefits to endurance athletes. Further research on the optimal aerobic training protocol performed with BFR is required to better improve endurance running performance and neuromuscular adaptations in elite runners.

### Practical applications

HIIT is a widely accepted training strategy for improving aerobic endurance performance. However, intense training programs may result in increases in catabolic hormonal responses, thereby reducing muscle mass and strength development after training. In addition, athletes often experience a rapid loss of training induced cardiorespiratory endurance and muscle strength adaptations because of insufficient training stimulus during off-season or unexpected situation. For example, the COVID-19 pandemic has prevented athletes from engaging in regular training, especially when lockdown measures are in place for ≥ 2 weeks, further reducing strength and conditioning performance. The results of this study indicated that 8 weeks of RIT-BFR is an effective method for improving RPmax, muscle strength, and muscle endurance. We recommend for coaches and endurance runners to use RIT (50% HRR, five 3-min sets, 1-min rest interval) combined with BFR (occlusion pressure: 1.3 × resting SBP) as part of a training strategy for promoting muscular fitness and endurance running performance and reducing the risk of detraining.
